# Understanding the Impedance Response of Lithium Polysulfide Symmetric Cells

**DOI:** 10.1002/smsc.202100042

**Published:** 2021-08-20

**Authors:** Yun-Wei Song, Yan-Qi Peng, Meng Zhao, Yang Lu, Jia-Ning Liu, Bo-Quan Li, Qiang Zhang

**Affiliations:** ^1^ Beijing Key Laboratory of Green Chemical Reaction Engineering and Technology Department of Chemical Engineering Tsinghua University Beijing 100084 China; ^2^ School of Materials Science and Engineering Beijing Institute of Technology Beijing 100081 China; ^3^ Advanced Research Institute of Multidisciplinary Science Beijing Institute of Technology Beijing 100081 China

**Keywords:** electrochemical impedance spectroscopy, equivalent circuits, lithium polysulfides, lithium–sulfur batteries, symmetric cells

## Abstract

Lithium–sulfur (Li–S) batteries are highly considered for next‐generation energy storage due to their ultrahigh theoretical energy density of 2600 Wh kg^−1^. The conversion reactions between lithium polysulfides (LiPSs) constitute the core process in working Li–S batteries. Electrochemical impedance spectroscopy (EIS) analysis of LiPS symmetric cells is an effective tool to provide detailed information on the LiPS conversion reactions and direct further kinetic promotion. However, reasonable interpretation of the EIS responses is so far insufficiently addressed without a well‐defined equivalent circuit. Herein, a systematic analysis on the EIS responses of LiPS symmetric cells is conducted to provide a comprehensible equivalent circuit. Interfacial contact, surface reaction, and diffusion are decoupled according to their respective characteristic frequency using the distribution of relaxation time analysis method. A well‐defined equivalent circuit is proposed to accurately fit the experimental EIS responses, unambiguously interpret key parameters, and be feasible with a wide range of experimental conditions. This work presents the methodology of understanding the EIS responses of LiPS symmetric cells and inspires analogous analysis on vital electrochemical processes.

## Graphical abstract

A systematic analysis on the impedance responses of lithium polysulfide symmetric cells is conducted. Contact effect, surface reaction, and diffusion are decoupled according to the distribution of relaxation time analysis. A well‐defined equivalent circuit is proposed and demonstrates feasibility and efficiency to accurately fit the experimental data and obtain key kinetic parameters.

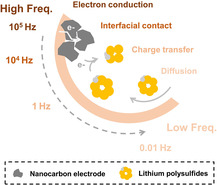

## 1 Introduction

Lithium–sulfur (Li–S) batteries are highly regarded as one of the most promising candidates for next‐generation energy‐storage systems.^[^ ^1^ ^]^ A 16‐electron reaction between sulfur (S_8_) and lithium (Li) promises an ultrahigh theoretical energy density of 2600 Wh kg^−**1**^.^[^ ^2^ ^]^ However, the Li–S electrochemical process goes through a complicated route from solid S_8_ to solid lithium sulfide (Li_2_S) with multiple dissolved lithium polysulfides (LiPSs) as vital intermediates.^[^ ^3^ ^]^ The LiPS‐mediated solid–liquid–solid conversion reactions are severely plagued by sluggish redox kinetics and therefore strongly limit the specific capacity, rate capability, and cycling stability of practical Li–S batteries.^[^ ^4^ ^]^ Various kinetic promotion strategies toward the polysulfide conversion reactions have been proposed to address the above issue. Chemical adsorption to immobilize the dissolved LiPSs,^[^ ^5^ ^]^ electrocatalysis on the LiPS conversion reactions,^[^ ^6^ ^]^ redox mediation and comediation on LiPS redox reactions,^[^ ^7^ ^]^ and regulation of the LiPS solvation structures^[^ ^8^ ^]^ have been confirmed to promote the polysulfide conversion kinetics and are widely used to enhance the battery performances.

Despite the proposed various strategies, a fundamental understanding on the mechanism of LiPS conversion reactions is still inadequate.^[^ ^9^ ^]^ Electrochemical impedance spectroscopy (EIS) is an effective tool to analyze complex LiPS conversion reactions with the advantages of high accuracy and rich information.^[^ ^10^ ^]^ By applying an alternating current (AC) perturbation to the electrochemical system and detecting the output signal in a wide frequency range, the involved electrochemical processes will exhibit characteristic impedance responses according to their typical relaxation time scales.^[^ ^11^ ^]^ Therefore, EIS analysis is able to decouple complicated electrochemical processes and reveal kinetic information.^[^ ^12^ ^]^ Meanwhile, symmetric cells with two identical nanocarbon‐coated metal foils as electrodes and LiPS‐containing electrolytes are the most commonly used cell configurations for EIS analysis. The dissolved LiPSs directly serve as the electrochemically active species and there is no need for lithium metal anode. Therefore, the interference from the anode side can be eliminated to endow an independent study on the cathodic LiPS conversion reactions.^[^ ^13^ ^]^ Several pioneer works have demonstrated the effectiveness of EIS analysis on LiPS symmetric cells regarding calibrating the kinetic parameters,^[^ ^14^ ^]^ probing the sulfur redox mechanism,^[^ 13, 15 ^]^ and evaluating the kinetics on different electrocatalysts.^[^ ^16^ ^]^ For instance, Duan and coworkers used LiPS symmetric cells with different heteroatom‐doped graphene electrodes as a model system to extract the activation energy of LiPS conversion by impedance tests at varied temperatures.^[^ ^17^ ^]^ The effect of N, S codoping on promoting the sulfur reduction reaction kinetics is accordingly confirmed.

Despite the earlier advantages, interpreting the impedance responses remains sophisticated due to the lack of a well‐defined analysis methodology. Surface reactions and diffusion are strongly coupled within the porous nanocarbon‐based electrodes to display complicated EIS spectra.^[^ ^18^ ^]^ Designing equivalent circuits to analyze the EIS spectra is widely used to interpret the impedance responses, where each equivalent element simulates a kinetic parameter of a specific electrochemical process.^[^ ^19^ ^]^ However, most of the equivalent elements fail to provide a verified explanation for the underlying physical meaning of the specific electrochemical process.^[^ ^20^ ^]^ Resistor–Capacitor (*R*–*C*) parallel circuits are optionally combined serially or parallelly to generate an equivalent circuit in most cases regardless of a reasonable physical meaning,^[^ ^21^ ^]^ and the simulated kinetic parameters are therefore ambiguous and cannot afford an accurate interpretation of the impedance responses of the LiPS symmetric cells. Talian et al. reported a transmission‐line model‐based grid circuit model from the analytical Newman's model.^[^ ^22^ ^]^ This work provides a general and accurate model to successfully simulate the impedance response of LiPS symmetric cells. Nevertheless, a brief, efficient, and comprehensible model is still needed for data fitting and analyzing.

**Figure 1:**
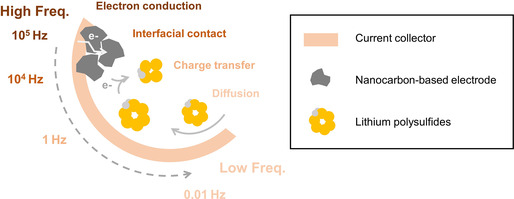
Schematic illustration of the impedance responses of LiPS symmetric cells according to the characteristic frequency.

In this contribution, a systematic interpretation of the EIS spectra of LiPS symmetric cells is conducted and an efficient and well‐defined equivalent circuit is proposed to obtain the key kinetic parameters. Distribution of relaxation time (DRT) analysis is first conducted to decouple the characteristic processes involved in a typical LiPS symmetric cell, including interfacial contact between the nanocarbon particles, surface charge transfer, and mass diffusion in the electrolyte following their respective characteristic frequency (**Figure** **1**). An equivalent circuit model based on the rational description of each process is then derived that exhibits good accuracy to describe the experimental EIS responses and effectively simulates key kinetic parameters of charge transfer resistance, double‐layer capacitance, diffusion impedance, and contact resistance in a wide range of experimental conditions. The well‐defined equivalent circuit can serve as an effective tool to analyze the impedance responses of LiPS symmetric cells for future researches.

## 2 Results and Discussion

**Figure 2:**
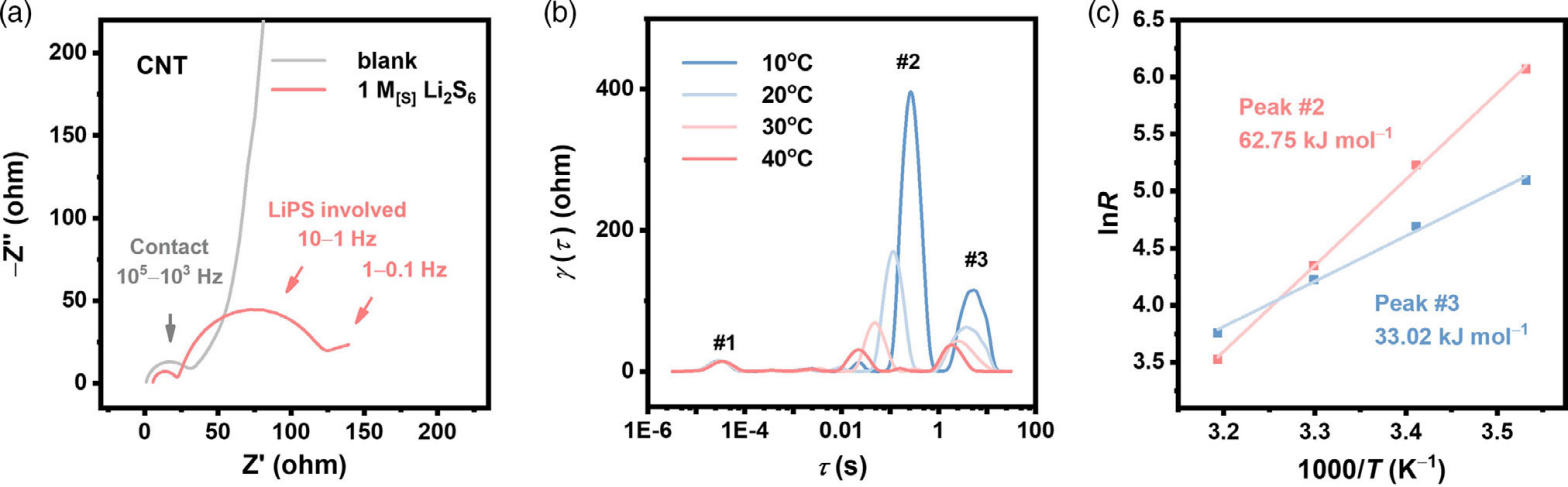
Decoupling the impedance responses of LiPS symmetric cells. a) Nyquist plot of symmetric cells with 1 M_[S]_ Li_2_S_6_ electrolyte or blank electrolyte. b) DRT analysis of the LiPS symmetric cells at different temperatures and c) corresponding Arrhenius behavior of the peaks in the DRT spectra.

EIS measurements with LiPS electrolyte (containing 1.0 M_[S]_ Li_2_S_6_) and blank electrolyte (containing no LiPS) were first conducted. Multiwalled carbon nanotube‐coated Al foils (named as CNT) were used as the electrodes. One small semicircle with a diameter of ≈15 ohm in the high frequency range (10^5^–10^3^ Hz), one larger semicircle of ≈50 ohm in middle frequency region (10–1 Hz), and an arc tail in the low frequency region to 0.1 Hz are clearly shown in the Nyquist plot of the symmetric cell with the LiPS electrolyte (**Figure** **2**a, red line). In contrast, the EIS spectrum of the blank electrolyte (gray line) similarly includes a semicircle at the high frequency region of 10^5^–10^3^ Hz with similar diameter but shows a 45° line and a near‐vertical line at the middle‐ and low frequency range, respectively. The above results indicate that the impedance responses in the medium and low frequency regions are responsible for LiPS conversion reactions, whereas the semicircle in the high frequency region is irrelevant to LiPS.

The electrochemical process can be experimentally distinguished according to their temperature dependence. EIS measurements of symmetric cells with the LiPS electrolyte in a wide temperature range were carried out to obtain the impedance response at different temperatures. Similar three‐arc behaviors are observed in the Nyquist plot at all temperatures (Figure S1, Supporting Information). The diameter of the semicircle in the middle frequency region is significantly reduced with elevated temperature, whereas the semicircle in the high frequency region is steady. To get a deeper understanding on the impedance responses, DRT analysis of the obtained impedance data was conducted. DRT analysis provides the impedance information of a system in the time domain and is able to distinguish the electrochemical processes with similar time constants which often overlap in the Nyquist or Bode plots.^[^ ^23^ ^]^ In brief, one electrochemical process is typically represented as a peak on the DRT spectra, where the integral area indicates the resistance of the process and the peak position represents its characteristic relaxation time.

As shown in the DRT spectra, three relaxation processes exhibited as three peaks can be observed at all temperatures (Figure 2b). The temperature dependence of these peaks is further revealed. Evidently, peak #1 at the lowest relaxation time (corresponding to the highest frequency region in the Nyquist plot) remains unchanged with varied temperatures, indicating that the process is irrelevant to electrochemical reaction or mass transport. Based on previous researches, the semicircle at the high frequency region in Figure 2a is attributed to the contact effects within the nanocarbon electrode, which results from the local resistance and capacitance when electrons are transmitted through the interface between nanocarbon particles.^[^ ^24^ ^]^ Meanwhile, peak #2 and peak #3 exhibit an Arrhenius relationship between resistance and temperature, as shown in Figure 2c. Peak #2 is located in the middle‐relaxation time range (0.01 − 1 s) to indicate a charge transfer behavior.^23^, ^25^ A large activation energy (*E*_a_) of 62.75 kJ mol^−1^ is observed, which is in agreement with the previously reported *E*_a_ values of the LiPS charge transfer processes.^[^ ^17^ ^]^ Similarly, peak #3 with the largest relaxation time can be considered as Warburg impedance caused by the diffusion of active materials in the electrolyte by comparing the *E*_a_ of 33.02 kJ mol^−1^ with the previously reported values of ion conduction in liquid electrolyte.^[^ ^26^ ^]^ Through matching the characteristic frequency (*f* = 1/*τ*) of the peaks in the DRT spectra with the frequency ranges in the EIS spectra, it is identified that the arcs of the Nyquist plot in the high, medium, and low frequency regions stem from the contact effects, LiPS surface reaction, and diffusion, respectively.

**Figure 3:**
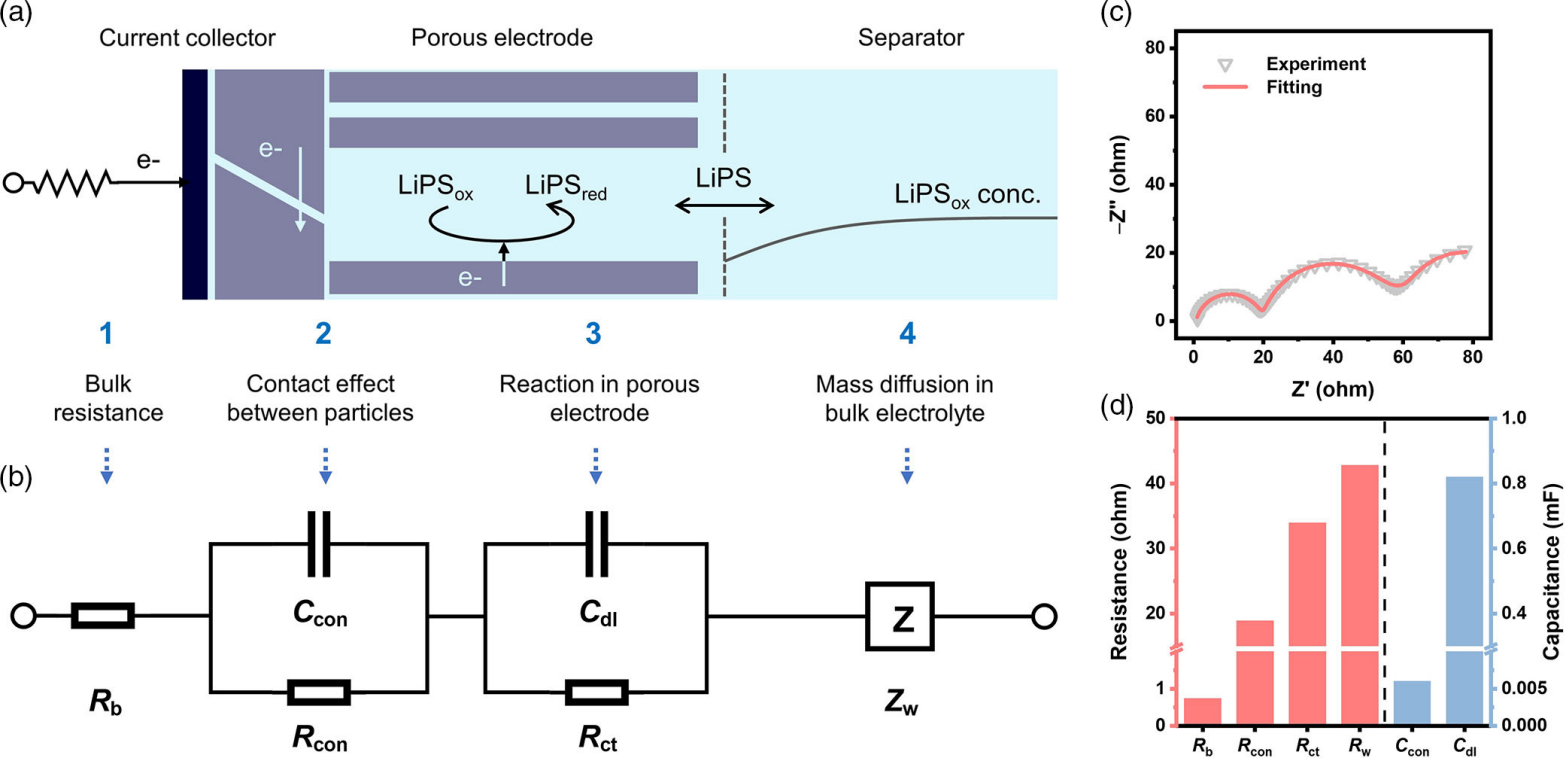
Proposing an equivalent circuit model to describe the impedance responses of LiPS symmetric cells. a) Characteristic processes involved in a practical LiPS symmetric cell and b) the corresponding equivalent circuit model. c) EIS experimental data and fitting results of a LiPS symmetric cell tested at 40 °C and d) key kinetic parameters interpreted using the proposed equivalent circuit model.

Based on the above experiments, four essential processes are identified and an equivalent circuit model is accordingly derived for interpretation of the EIS results (**Figure** **3**a,b). First of all, a resistor, *R*_b_, is selected to represent the bulk resistance of an electrochemical system, including ion migration resistance of bulk electrolyte and ohmic resistance of the current collector that do not produce capacitive responses. Next, the contact effect dominant in the high frequency region is modeled through a parallel circuit of *R*_con_ and *C*_con_, corresponding to the contact resistance and the contact capacitance between the nanocarbon particles and between the nanocarbon particles and the current collector.

The description of surface reactions in the porous electrode is more complicated. Concretely, the CNT electrode exhibits a dense but porous morphology with twisted and crosslinked pores (Figure S2, Supporting Information). During the EIS test, the LiPS conversion reactions take place on the conductive surfaces of CNTs, whereas the mass transport resistance from the bulk electrolyte to the reactive surfaces varies at different depths along with the pores, leading to dispersed impedance responses across the porous electrode. To describe the earlier process, a modified transmission‐line model (the so‐called de levie element) is introduced to simulate the complex reaction of LiPS within the porous electrode.^[^ ^27^ ^]^ As shown in Figure S3, Supporting Information, the transmission‐line element considers the electrode interface within the pores as a series of parallelly connected microregions. Each microregion consists of a parallel circuit of differential double‐layer capacitance (*C*_dl_/*n*) and charge transfer impedance (*n*·*R*_ct_), corresponding to the LiPS conversion reactions. An upper rail of the resistors (*R*_EI_/*n*) and a lower rail of the resistors (*R*_ion_/*n*) between each parallel circuit account for the electron conduction and ion migration resistance across the electrolyte within the porous electrode, respectively. The concentration gradient of LiPSs within the pores is not considered for the LiPS redox reactions taking place simultaneously across the pores to render a uniform concentration distribution. To simplify, *R*_EI_ is neglected as the conductivity of nanocarbon materials is much higher than the ionic conductivity in the electrolyte. Accordingly, the total impedance of the transmission‐line element is described by the following equation.

(1) $$Z_{\text{TL}}=\sqrt{R_{\text{ion}}Z_{s}}\coth(\sqrt{R_{\text{ion}}/Z_{s}})$$

(2) $$Z_{s}=\frac{R_{\text{ct}}}{1+j\omega R_{\text{ct}}C_{\text{dl}}}$$

where *Z*_TL_ is the total impedance of the transmission‐line element, *Z*_s_ is the impedance of LiPS surface reaction, *j* is the imaginary unit, and *ω* is the angular frequency of the EIS test. When *R*_ion_ is much smaller compared with the *R*_ct_, the model can be further simplified to an *R*–*C* parallel circuit as

(3) $$Z_{\text{TL}}=Z_{s}$$

The above hypothesis is reasonable, given the sluggish kinetics of the polysulfide reaction under practical testing conditions, which accords with the general understanding and is verified by the subsequent fitting results. Therefore, an *R*–*C* parallel circuit can be directly used herein to describe the LiPS conversion reactions in the porous electrode in cases of small *R*_EI_ and *R*_ion_.

As the reaction occurs symmetrically on both sides of the cell, the concentration value at the center point of the separator will be constant (i.e., a finite‐length boundary layer). Therefore, a finite‐length Warburg component *Z*_w_ to describe the diffusion of LiPSs in the bulk electrolyte is introduced in the main circuit. For the reaction

(4) $$p{\text{LiPS}}_{\text{Ox}}+ne^{-}+n{\text{Li}}^{+}=q{\text{LiPS}}_{\text{Red}}$$

there is

(5) $$Z_{w}=R_{w}\frac{\tanh\left(\sqrt{j\omega L^{2}/D_{\text{LiPS}}}\right)}{\sqrt{j\omega L^{2}/D_{\text{LiPS}}}}$$

(6) $$R_{w}=\frac{RT}{n^{2}F^{2}}\frac{L}{AD_{\text{LiPS}}}(\frac{p}{C_{{\text{LiPS}}_{\text{Red}}}}+\frac{q}{C_{{\text{LiPS}}_{\text{Ox}}}})$$

where *R* is the ideal gas constant, *T* is the temperature, *F* is Faraday's constant, *L* is the thickness of diffusion boundary layer, *A* is the cross‐sectional area of the diffusion route, *n* is the electron transfer number, *D*_LiPS_ is the diffusion coefficient of LiPS (the value is assumed to be equal for LiPS_Red_ and LiPS_Ox_), $C_{{\text{LiPS}}_{\text{Red}}}$and $C_{{\text{LiPS}}_{\text{Ox}}}$ are the bulk concentrations of the LiPSs, and *p* and *q* are the stoichiometric numbers of the LiPS conversion reaction. As defined in the earlier equation, $R_{w}$ is the lumped diffusion resistance. Under the same experimental conditions, $R_{w}$ is inversely proportional to the diffusion coefficient. Therefore, the proposed equivalent circuit of the electrochemical system is finally shown in Figure 3b. To manifest the rationality of the simplification and validity of the model, the proposed equivalent circuit is used to fit the experimental data of a LiPS symmetric cell tested at different temperatures and obtain the kinetic parameters. A comparison between the experimental and fitted EIS measurements in Nyquist plots is shown in Figure 3c and S4, Supporting Information. The good agreement demonstrates that the proposed model can effectively describe the impedance responses of practical LiPS symmetric cells.

Based on the earlier fitting results, several key kinetic parameters of the LiPS symmetric cells are determined by the proposed model (Figure 3d). The bulk resistance is 0.73 ohm, indicating a negligible resistance of ion conduction in the bulk electrolyte. A considerable contact resistance of 18.87 ohm and a small contact capacitance of 6.30 μF confirm the poor contact between carbon nanoparticles (small contact area leads to large contact resistance). The charge transfer resistance of 33.95 ohm and the lumped diffusion impedance of 42.78 ohm are within reasonable ranges, implying that the diffusion impedance becomes dominant at higher temperatures. The double‐layer capacitance exhibits a large value of 0.82 mF, indicating the porous nature of the nanocarbon electrode. Further, the fitting results of the transmission‐line model and the simplified *RC* circuit model are compared (Figure S5, Supporting Information). It can be noticed that even the cell is tested at 40 °C with a relatively lower *R*_ct_, the value of *R*_ion_ is 9.5 ohm and still small enough to be neglected. Also, the other fitting results do not have a significant difference. This indicates that the simplification herein is reasonable and manifests the effectiveness of the proposed model.

**Figure 4:**
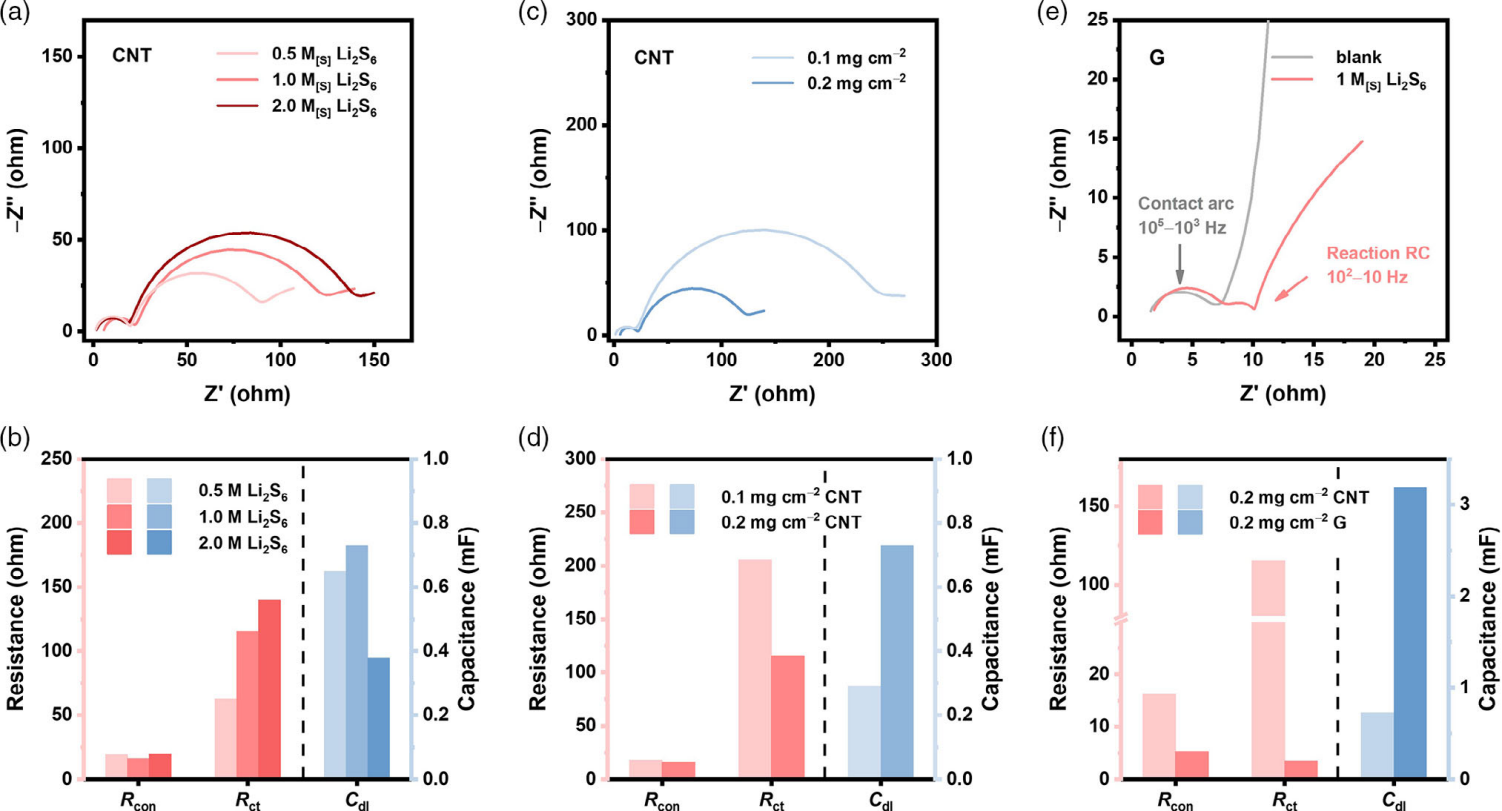
Applicability of the proposed equivalent circuit model in a wide range of experimental conditions. EIS spectra and fitting results of a,b) electrolyte with different concentrations of Li_2_S_6_, c,d) electrode with different mass loadings of CNT, and e,f) electrode with different types of nanocarbon in Li_2_S_6_ and blank electrolyte.

To further explore the applicability of the earlier analysis method, the proposed model is further applied to a wide range of experimental conditions. EIS spectra of LiPS symmetric cells with different concentrations of LiPS in the electrolyte, different areal loadings of nanocarbon in the electrode, and different types of nanocarbon in the electrode are measured. The corresponding kinetic parameters are fit using the proposed model. First, the effect of LiPS concentration on impedance response is studied. As the Li_2_S_6_ concentration increases, *R*_con_ remains at the same level to prove that *R*_con_ is only related to the contact state between the nanocarbon particles. In contrast, *R*_ct_ shows an increased value of 62.8, 115.5, and 139.6 ohm with 0.5, 1.0, and 2.0 M_[S]_ Li_2_S_6_, respectively (**Figure** **4**a,b, and S6, Supporting Information). *C*_dl_ also significantly decreases at high concentrations. This is consistent with the deterioration of reaction kinetics at low electrolyte to sulfur ratios and indicates that the properties of the electric double layer may change at higher LiPS concentrations.^[^ ^28^ ^]^

Electrodes with different areal loadings of 0.1 and 0.2 mg cm^−2^ CNTs were then applied in LiPS symmetric cells to study the influence of electrode thickness. As the areal loading increases from 0.1 to 0.2 mg cm^−2^, the active surface area of the electrode shall also be doubled. Accordingly, the *R*_ct_ is reduced from 205.5 to 115.5 ohm and the *C*_dl_ nearly doubles from 0.29 to 0.73 mF consistently (Figure 4c,d). Meanwhile, *R*_con_ is constant as the contact state between the nanocarbon particles remains unchanged, indicating that the attribution of the high‐frequency semicircle to the contact resistance is reasonable.

Finally, a graphene‐coated electrode (named as G) is substituted for the CNT electrode to probe the effect of the electrode materials. As shown in Figure S7, Supporting Information, the G electrode exhibits a more loose packing structure to endow effective exposure of the active surfaces. Figure 4e shows the EIS spectra of the symmetric cells based on the G electrode. A similar behavior including two semicircles with an arc tail is observed as the CNT electrode. However, the overall impedance of the G electrode is significantly lower than the CNT electrode (Figure 4f and S8, Supporting Information). *R*_con_ decreases from 17.9 to 5.3 ohm and *R*_ct_ shows a dramatic drop from 115.5 to 3.5 ohm. This can be explained by the improved contact state (from point−point contact to surface−surface contact) and increased specific surface area of G. Electrochemical active surface area (ECSA) measurements are further carried out to verify the fitting results. As shown in Figure S9, Supporting Information, the double‐layer capacitance of G and CNT measured by cyclic voltammetry is 3.07 and 0.89 mF, respectively, which are very close to the fitted value of 3.19 and 0.73 mF, respectively, indicating that the proposed model can unambiguously interpret key kinetic parameters in LiPS symmetric cells.

The earlier discussion manifests the wide applicability of the proposed equivalent circuit model and encourages the extension of this research paradigm into other electrochemical systems. Specifically, different characteristic processes can be decoupled and identified by DRT analysis from the experimental impedance response of an electrochemical system. Then, a well‐defined equivalent circuit model shall be constructed accordingly. After that, subsequent tests with a wide range of experimental conditions are expected to demonstrate the feasibility of interpreting the key kinetic parameters. Notably, the selection of suitable equivalent elements must depend on the property of the electrochemical system. For instance, a transmission‐line circuit has to be employed to describe the reaction in a porous electrode when *R*_ion_ is non‐negligible compared with *R*_ct_. Otherwise, a simplified *RC* parallel circuit can be used instead. Furthermore, the well‐defined equivalent circuit model enables us to evaluate a specific electrocatalyst or sulfur cathode by EIS method through interpreting the key kinetic parameters unambiguously so as to find the direction for further improvements. For instance, the charge transfer resistance *R*_ct_ reflects the intrinsic activity of the electrocatalyst in the electrode to serve as an indicator. When *R*_con_ is found to be too large for an electrode, the introduction of additional conductive agent can effectively improve the overall performance. When *R*_w_ prevails, reducing the electrolyte viscosity or using polar sites to anchor the LiPSs will be helpful.

## 3 Conclusion

To summarize, this work conducts an in‐depth analysis of the EIS response of LiPS symmetric cells to provide a well‐defined equivalent circuit model. DRT analysis reveals that the impedance responses can be decoupled into three relaxation processes including the contact effect between nanocarbon particles, charge transfer at active surfaces, and mass diffusion in the electrolyte. A reliable equivalent circuit is then proposed accordingly and shows feasibility and efficiency to accurately fit the experimental data and obtain key kinetic parameters including charge transfer resistance, double‐layer capacitance, and diffusion impedance with a wide range of experimental conditions. This work not only presents a comprehensive methodology to interpret the EIS responses of LiPS symmetric cells but also provides a general research paradigm for the study of parallel electrochemical systems.

## Conflict of Interest

The authors declare no conflict of interest.

## Data Availability Statement

Data available on request from the authors.

## Supporting information

Supplementary Material
