# Supplementary material for: Understanding the Impedance Response of Lithium Polysulfide Symmetric Cells
Source: Small Sci. 2021 Aug 20;1(11):2100042. doi: 10.1002/smsc.202100042 (PMC11935945; doi:10.1002/smsc.202100042)
Supplement: Supplementary file 1 — Supplementary Material [file SMSC-1-2100042-s001.pdf]

1. Experimental section

1.1. Materials

All the raw materials, including sulfur (S), lithium sulfides (Li_2S), bis(trifluoromethane)sulfonamide lithium salt (LiTFSI), 1,2-dimethoxyethane (DME), 1,3-dioxolane (DOL), lithium nitrate ($LiNO_3$), N-methyl pyrrolidinone (NMP), deionized water (DIW), Celgard 2400 polypropylene (PP) membranes, multi-walled carbon nanotube (CNT), graphene (G), LA132 binder, polyvinylidene fluoride (PVDF, HSV-300), and aluminum (Al) foil were purchased from commercial sources and directly used without further purification.

1.2. Preparation of nanocarbon-coated Al foil

For the CNT-coated Al foil，90 wt.% CNT and 10 wt.% LA132 binder were homogenized and dispersed in DIW to form a dilute slurry. The prepared CNT slurry was subsequently coated on Al foils, dried at 60°C for 30 minutes, and punched into disks with a diameter of 13.0 mm as electrodes. The areal loadings of CNT were controlled as 0.10 and 0.20 mg cm^{-2} for the measurements, respectively.

For the G-coated Al foil, 90 wt.% G and 10 wt.% PVDF were ultrasonically dispersed in NMP to form a dilute slurry. The prepared G slurry was subsequently coated on Al foils, dried at 60°C for 30 minutes, and punched into disks with a diameter of 13.0 mm as electrodes. The areal loading of G was controlled as 0.20 mg cm^{-2} for the measurements.

1.3. Electrolyte and polysulfide preparation

1.0 mol L^{-1} LiTFSI dissolved in DOL/DME (v/v = 1:1) solvents was adopted as the blank electrolyte. Li_2S_6 was selected as the prototype of polysulfides. The mixture of Li_2S and S with a molar ratio of 1:5 was added into the blank electrolyte to prepare Li_2S_6 (0.5, 1.0, and 2.0 mol L^{-1} [S]) catholyte. All the above procedures were conducted in an Ar-filled glove box.

1.4. Cell assembly and electrochemical evaluation

Symmetric cells were assembled into a standard 2025 coin cell configuration. Both the electrodes of the symmetric cells adopted the nanocarbon-coated Al foils (CNT or G). The separators were Celgard 2400 PP membranes with a diameter of 19.0 mm. The amount of electrolyte was 40 μL for each cell.

Electrochemical impedance spectroscopy (EIS) and electrochemical active surface area (ECSA) analysis were conducted on a Solartron 1470E electrochemical workstation. EIS was performed using the potentiostat mode at the open-circuit voltage. A sinusoidal voltage with an amplitude of 10 mV and a frequency range from 0.1 Hz to 10 kHz was applied. A high and low temperature alternating test chamber (WGD6005) was used in conjunction with the Solartron electrochemical workstation to measure the impedance from 0°C to 10°C, 20°C, 30°C, and 40°C, respectively. The interval time between each temperature was 10 min. Distribution of relaxation time (DRT) analysis was conducted by applying the MATLAB GUI based DRTtools which are devoloped by Ciucci’s groups.[1] The paremeters for DRT is based on the Gaussian

discretization method with a regularization parameter of 10^{-4} and FWHM control of 0.5, where the inductive data was neglected during fitting. ECSA analysis was performed at a scan rate of 10, 20, 40, 60, 80, and 100 mV s^{-1} between −0.05 and 0.05 V, and the linear relationship between the measured current densities and the scan rates is used to fit the double layer capacitance.

1.5. Material characterization

The morphologies and thickness of nanocarbon-coated Al foils were characterized by a JSM 7401F (JEOL Ltd., Tokyo, Japan) scanning electron microscopy (SEM) operated at 3.0 kV.

2. Supporting figures

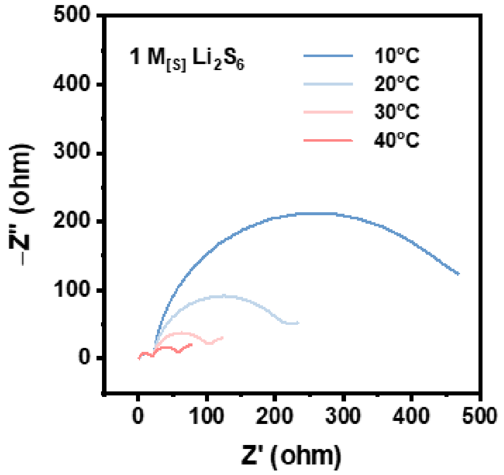

Figure S1. EIS spectra for LiPS symmetric cells at varied temperatures.

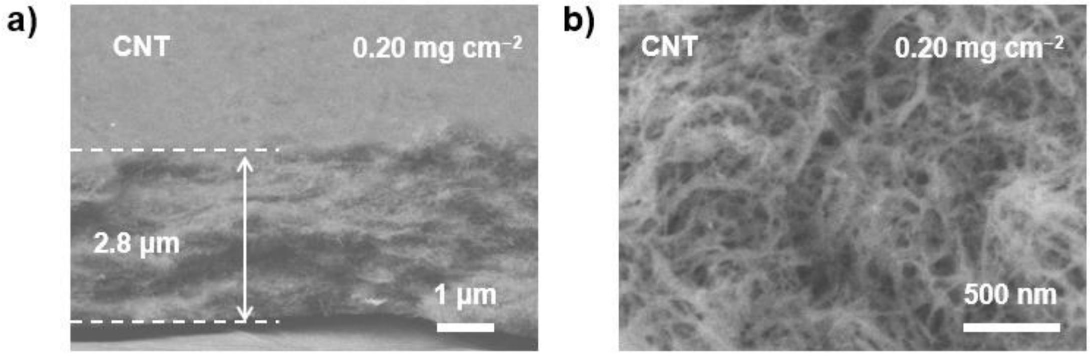

Figure S2. SEM images of CNT-coated Al foil from a) side view and b) top view. The areal loading of CNT was 0.20 mg cm^{-2}.

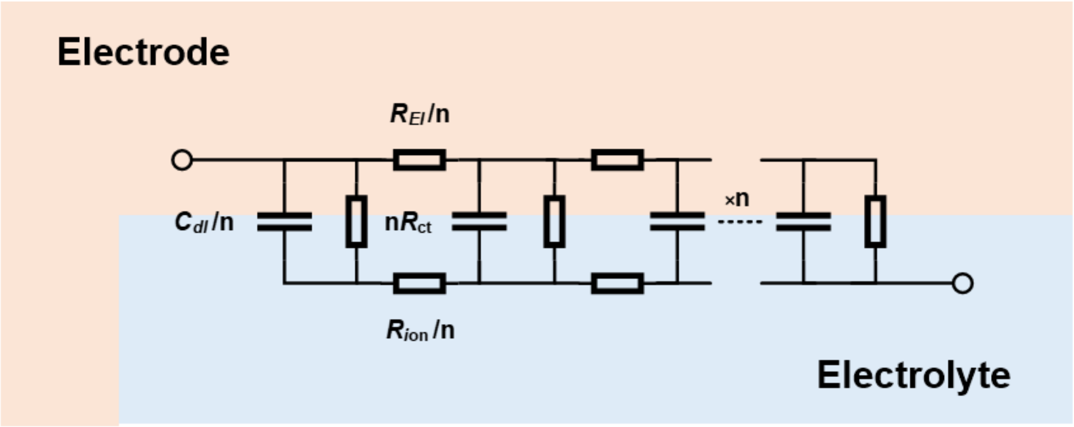

Figure S3. Scheme of the transmission line model and corresponding equivalent circuit to describe the impedance responses within porous electrodes.

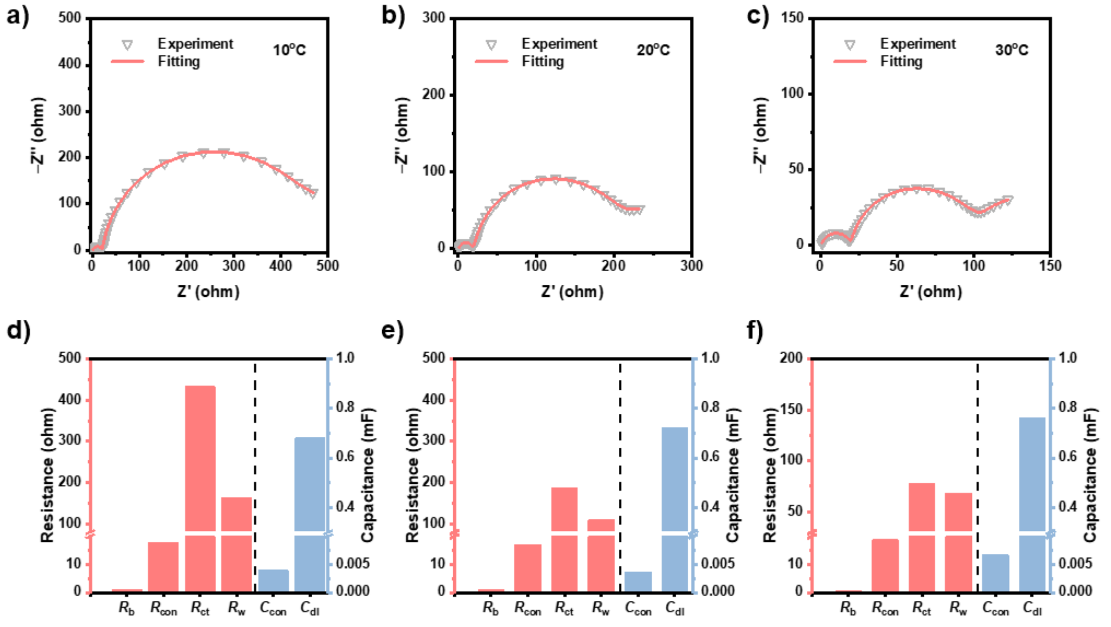

Figure S4. EIS experimental data and fitting results of LiPS symmetric cells tested at a) 10°C, b) 20°C, and c) 30°C. d−f) Corresponding key kinetic parameters obtained using the proposed equivalent circuit model.

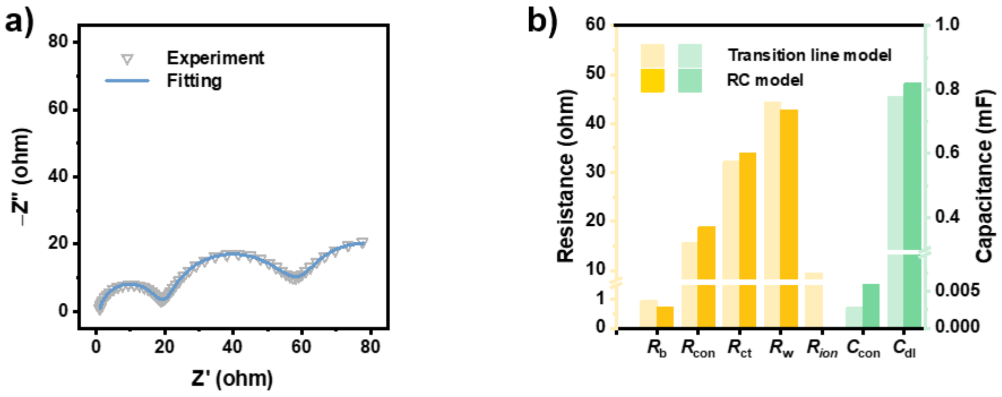

Figure S5. Comparation of the transmission line model and the simplified RC model by a) fitting a practical EIS spectra tested at 40°C and b) comparing the obtained kinetic parameters.

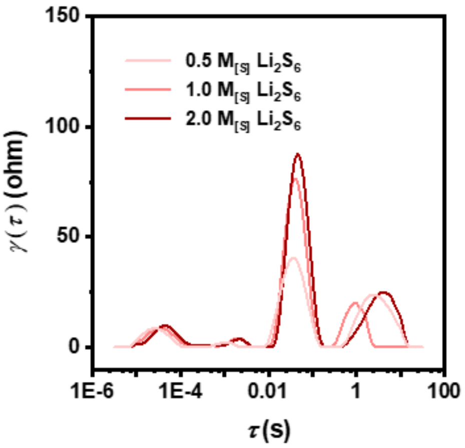

Figure S6. DRT analysis results of the LiPS symmetric cells with varied polysulfide concentrations.

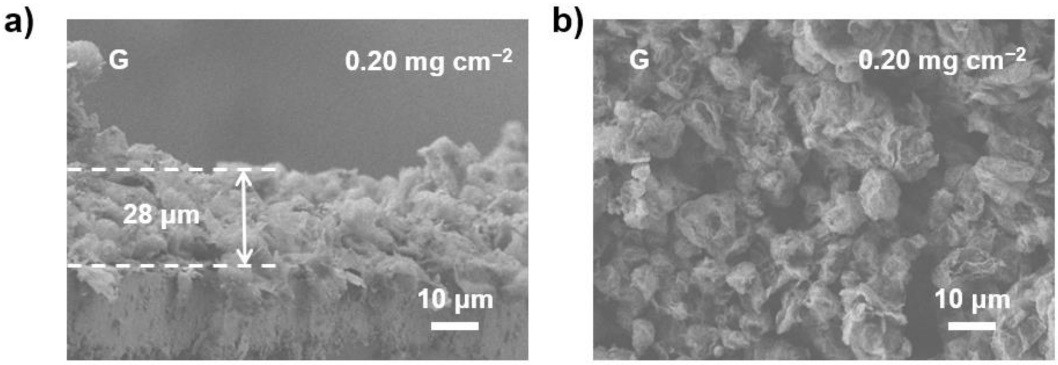

Figure S7. SEM images of G-coated Al foil from a) side view and b) top view. The areal loading of G was 0.20 mg cm^{-2}.

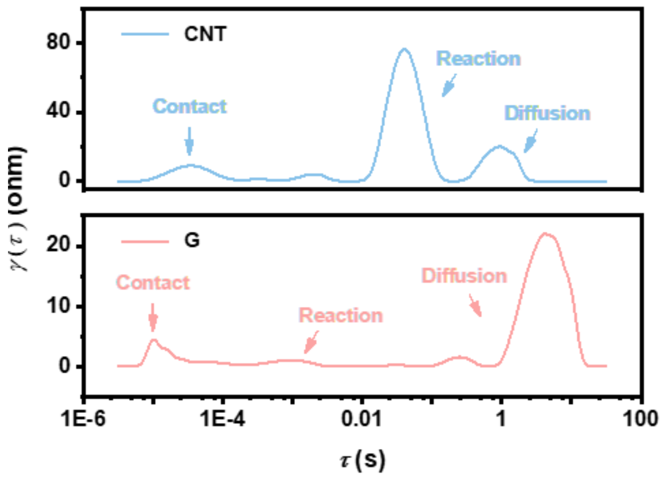

Figure S8. DRT analysis results of the LiPS symmetric cells with different nanocarbons.

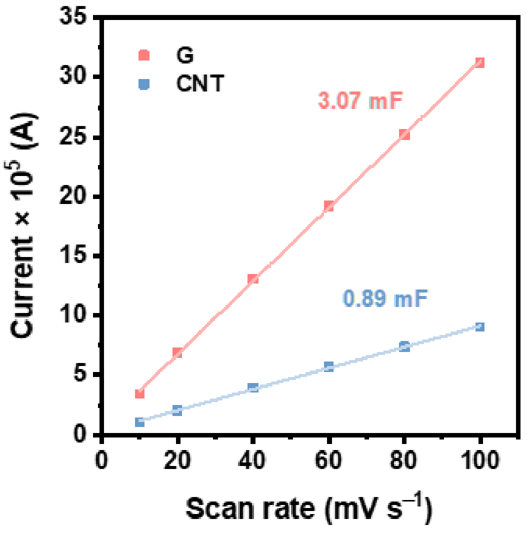

Figure S9. ECSA measurements of G and CNT to determine the electric double layer capacitance.

3. Supporting references

[1] T. H. Wan, M. Saccoccio, C. Chen, F. Ciucci, *Electrochim. Acta* **2015**, *184*, 483.
